# Tunnel/Layer Composite Na_0.44_MnO_2_ Cathode Material with Enhanced Structural Stability via Cobalt Doping for Sodium-Ion Batteries

**DOI:** 10.1021/acsomega.3c02315

**Published:** 2023-07-22

**Authors:** Erdinc Oz, Serdar Altin, Sevda Avci

**Affiliations:** † Physics Department, Ataturk University, Erzurum 25400, Turkey; ‡ Nanoscience and Nanoengineering Department, Ataturk University, Erzurum 25400, Turkey; § Physics Department, Inonu University, Malatya 44210, Turkey; ∥ Department of Engineering Physics, Istanbul Medeniyet University, Istanbul 34700, Turkey

## Abstract

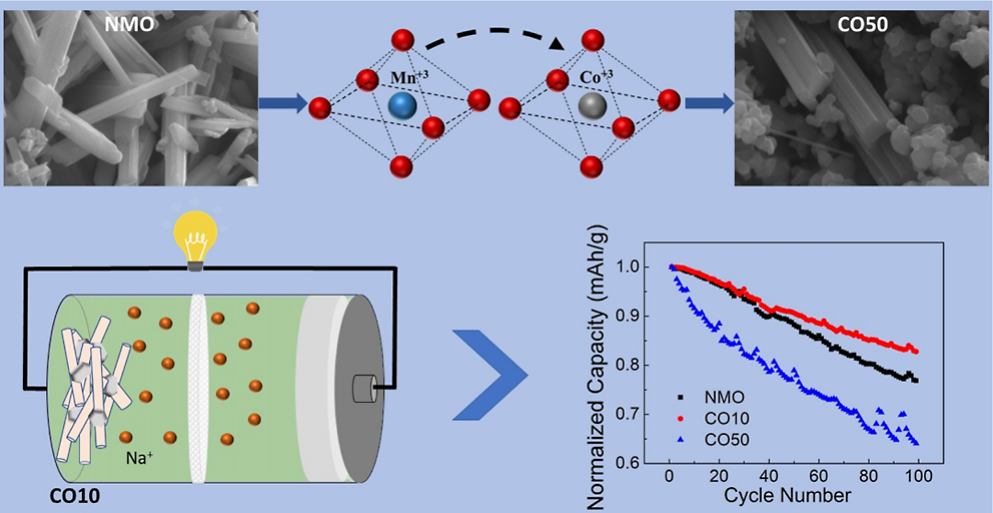

Sodium-ion batteries (SIBs) are the most promising alternative to lithium-ion batteries (LIBs) due to their low cost and environmental friendliness; therefore, enhancing the performance of SIBs’ components is crucial. Although most of the studies have focused on single-phase cathode electrodes, these materials have difficulty in meeting the requirements in practice. At this point, composite materials show superior performance due to balancing different structures and are offered as an alternative to single-phase cathodes. In this study, we synthesized a Na_0.44_MnO_2_/Na_0.7_MnO_2.05_ composite material in a single step with cobalt substitution. Changes in the crystal structure and the physical and electrochemical properties of the composite and bare structures were studied. We report that even if the initial capacity is slightly lower, the rate and cyclic performance of the 1% Co-substituted composite sample (CO10) are superior to the undoped Na_0.44_MnO_2_ (NMO) and 5% Co-substituted (CO50) samples after 100 cycles. The results show that with the composite cathode phase transformations are suppressed, structural degradation is prevented, and better battery performance is achieved.

## 1 Introduction

Increasing energy demand has motivated innovation in energy storage systems. Although the most popular batteries used in these systems are lithium-ion batteries (LIBs), the increase in cost in recent years has accelerated the search for alternative strategies.^1^ At this point, sodium-ion batteries (SIBs) are seen as the most suitable alternative. Because sodium is one of the most abundant elements in the earth’s crust,^2^ and aluminum foil, which costs one-third of copper foil, can be used as the anode,^3^ SIBs are less costly than lithium-based batteries. SIBs are also easy to develop, as their components and operating mechanism are the same as LIBs.^4^ In addition, SIBs, with their environmentally friendly nature, are the most suitable alternative to replace carbon-based energy sources, which is a common view that reducing their use is essential for sustainability.

On the other hand, there are also negative features of SIBs. Due to the larger size of Na^+^ than Li^+^, structural degradation occurs, especially in layered crystal structures, resulting in capacity losses during the charge/discharge process.^5,6^ Different methods, such as coating,^7,8^ nanometerization,^9,10^ etc., have been tried to overcome this structural degradation. Another major challenge is the irreversible phase transformations that occur during Na extraction. P2 → O2 phase transitions, especially in layered structures, cause rapid structural degradation resulting in capacity loss.^11^ One way to overcome this problem is to prevent phase transformations, usually occurring in the high-voltage region, by lowering the high operating voltage.^12,13^ But this implies that the performance of the battery is not fully utilized. Another way to achieve structural stabilization by preventing phase transition is by cation substitution to transition-metal sites and Na^+^ layers.^14−17^ Prakash et al. studied the substitution of Ni and Mg for Mn sites in Na_0.7_MnO_2_ cathode material with a P2 structure to eliminate the Jahn–Teller effect on Mn^3+^.^18^ They report an energy density of 335 W h kg^–1^ in the 1.5–4.2 V potential range in the Ni- and Mg-substituted P2-Na_0.67_Ni_0.25_Mg_0.1_Mn_0.65_O_2_ cathode. In another remarkable study, Wu, Geng, and Lü et al. obtained a multi-synergetic structure P2-Na_0.67_ [Li_0.1_ (Mn_0.7_Ni_0.2_Co_0.1_) _0.9_] O_2_ by substituting Ni, Co, and Li into Na_0·67_MnO_2_.^19^ Here, Ni and Co substitution for Mn sites prevented Jahn–Teller degradation on Mn, suppressed the phase transformation, and increased the structural stability, while Li substitution into metal oxide sites led to the formation of a ribbon superstructure. In electrochemical tests, a discharge capacity of 123.5 mA h g^–1^ was obtained at a current density of 10 mA g^–1^, and a capacity retention performance of 94.4% was obtained at the end of 100 cycles.

Na_0.44_MnO_2_ (NMO) is a promising cathode among NaMO_2_ (M = transition metal) materials and has a relatively high theoretical capacity (∼122 mA h g^–1^) in the 2.0–4.0 V potential window.^20−22^ The crystal structure of NMO consists of a large S-shape and a smaller pentagon tunnel formed by the combination of the MnO_5_ square pyramids and MnO_6_ octahedra. Half of the Mn^3+^ ions are in the MnO_5_ square pyramids, while the remaining half and all the Mn^4+^ ions are in the octahedral MnO_6_ structure.^23,24^ S-shaped tunnels, serving as fast ion diffusion pathways, are ideal for large Na^+^ ions.

Despite all these unique properties of NMO, the low ion kinetics and the large radius of sodium negatively affect the electrochemical performance. At this point, modifications to the morphology can shorten the diffusion path, increase the ion kinetics, and improve the electrochemical performance. One of the main factors affecting morphology is the synthesis method. Therefore, different synthesis techniques, such as solid-state,^25^ hydrothermal,^26^ sol–gel,^27^ and molten salt,^28^ have been used for NMO. Cao et al. report a 77% capacity retention at the end of 1000 cycles for Na_0.44_MnO_2_, which was synthesized in nanowire morphology by a polymer-pyrolysis method.^29^ Sodium-ion diffusion occurs through the large S-shaped tunnels along the *c*-axis; thus, the nano-wire structure’s elongation means an extension of the diffusion path. Zhou et al. synthesized Na_0.44_MnO_2_ in nanoplate morphology to shorten the diffusion path by reducing the nanowire length-to-radius ratio. They report a 122 mA h g^–1^ reversible capacity at 0.085 C rate and 90% capacity retention after 100 cycles at 1.14 C rate between 1.25 and 4.0 V.^30^

Another way to improve the performance is cation substitution for Mn sites.^31,32^ Chen et al. report a composite structure of NMO with high capacity by substituting Co into the Mn site.^33^ Upon increasing the Co content, P2 and P3 phases start to form in addition to the tunnel-type NMO parent structure, forming a composite material. While tunnel-type NMO has a rod-like morphology, layered P2 and P3 phases have nanoplate and granular morphologies. In electrochemical tests, the composite material reached an initial capacity of 220 mA h g^–1^ at 2.0–4.2 V with the C/10 rate and 104 mA h g^–1^ at 2.0–4.0 V with the 5 C rate.

In this article, we present the physical and electrochemical properties of Co-substituted Na_0.44_MnO_2_. The investigation was conducted with utmost attention to detail, and the results were thoroughly analyzed to reveal the impact of this substitution on the material’s electrochemical performance. The physical measurements show that the Co substitution for Mn sites creates a composite material upon formation of the P2 and P3 layered structures alongside the tunnel structure of NMO, creating a composite material. However, the Co ions tend to substitute into P2/P3 layered structures instead of the tunnel structure of NMO. Electrochemical performance tests demonstrate that 1% Co-substituted composite material is remarkably structurally stable compared to NMO with a tunnel structure.

## 2 Experimental Section

### 2.1 Material Preparation

The samples of Na_0.44_Mn_1–*x*_Co_*x*_O_2_ (*x* = 0, 0.01, 0.05) were synthesized by a simple solid-state reaction. Na_2_CO_3_ (Sigma, >99%), MnCO_3_ (Sigma, >99%), and Co_3_O_4_ (Sigma, >99%) raw materials were mixed in a stoichiometric ratio. An extra 10 wt % NaCO_2_ was added to the mixture to compensate for the loss of Na at high temperatures. Raw powders were mixed by ball-milling for 1 h to obtain a homogeneous mixture, then placed in an alumina boat and calcined at 300 °C for 8 h. The mixtures were heated at 800 °C for 9 h to obtain the final structure. All heat treatments were carried out in the air atmosphere with a 5 °C/min heating rate and cooled uncontrolled to room temperature. Due to the moisture sensitivity of the samples, all samples were placed in a glovebox filled with argon after heat treatment.

### 2.2 Material Characterization

High-resolution synchrotron powder diffraction data were collected at the P02.1 beamline at PETRA III (DESY, Hamburg). The wavelength was fixed at 0.2066 Å (∼60 keV) with a 60 s exposure time and a relative energy bandwidth Δ*E*/*E* of 10^–4^ during the experiment. Before data collection, the 1624 detector (PerkinElmer) system was calibrated using the LaB_6_ standard (NIST). During powder X-ray diffraction (XRD) measurements, to increase the data count, the samples were filled into Kapton capillary and rotated with the help of a stepper motor. The data were fitted by the Rietveld method using FullProf software.^34^

The morphology of the samples was analyzed by scanning electron microscopy (SEM) with an EVO 40 XVP (LEO) with 30 keV primary electron energy. Detailed crystal structure was investigated by transmission electron microscopy (TEM) with a high-resolution transmission electron microscope (JEM-2011). A Gatan CCD camera recorded the selected area electron diffraction (SAED) pattern. The Mn K-edge spectra were collected by X-ray absorption near-edge structure (XANES) and extended X-ray absorption fine structure (EXAFS) techniques at the P64 beamline Deutsches Elektronen-Synchrotron (DESY, PETRA III, Germany). All samples were mixed with cellulose and pelletized under 5 tons of pressure. All the Mn-K edge spectra were measured at room temperature and in fluorescence mode. Each XAFS measurement was repeated three times to obtain acceptable quality spectra and averaged. EXAFS data were extracted from XAS data and analyzed by ATHENA software.^35^ Raman spectra were measured using the Senterra microscope (RIGAKU) with a 532 nm excitation wavelength. Temperature-dependent magnetization (M–T) measurements were taken with a vibrating sample magnetometer attachment on a PPMS device (Quantum Design) under a magnetic field of 1000 Oe. The temperature was controlled between 5 and 300 K. Elemental analysis was performed using an inductively coupled plasma mass spectrometer (Agilent 7800).

### 2.3 Electrochemical Tests

For electrochemical tests, 80% active material, 10% carbon black, and 10% polyvinylidene fluoride were mixed, and *N*-methy1-2-pyrrolidone was added to turn the mixture into a slurry, which was then coated on an aluminum foil. The film was dried at 120 °C under vacuum overnight, then punched as 10 mm diameter disks for use as a working electrode. Working electrode disks were transferred to an argon-filled glovebox to make coin cells. The active material mass loadings of the electrodes were ∼3 mg.

Coin cells (CR2032 type) were assembled in an argon-filled glovebox (MBraun) with Na chips (12 mm dia.) as an anode, 250 μL of electrolyte [1 M NaClO_4_ in EC/PC (50:50, wt.)], and GF/D filter paper (Whatman) as a separator. The charge–discharge test was performed in the 2.0–4.0 V voltage range with constant current technique at room temperature with an OctoStat (IviumStat) workstation. The rate capability tests were obtained at different current ranges from 0.2 to 2 C (1 C = 121 mA h/g) between 2.0 and 4.0 V. Cyclic voltammetry (CV) measurements were performed on the electrochemical workstation (VMP3, BioLogic) at a scanning rate of 0.1 mV/s and a potential range of 2.0–4.0 V.

## 3 Results and Discussion

**Figure 1:**
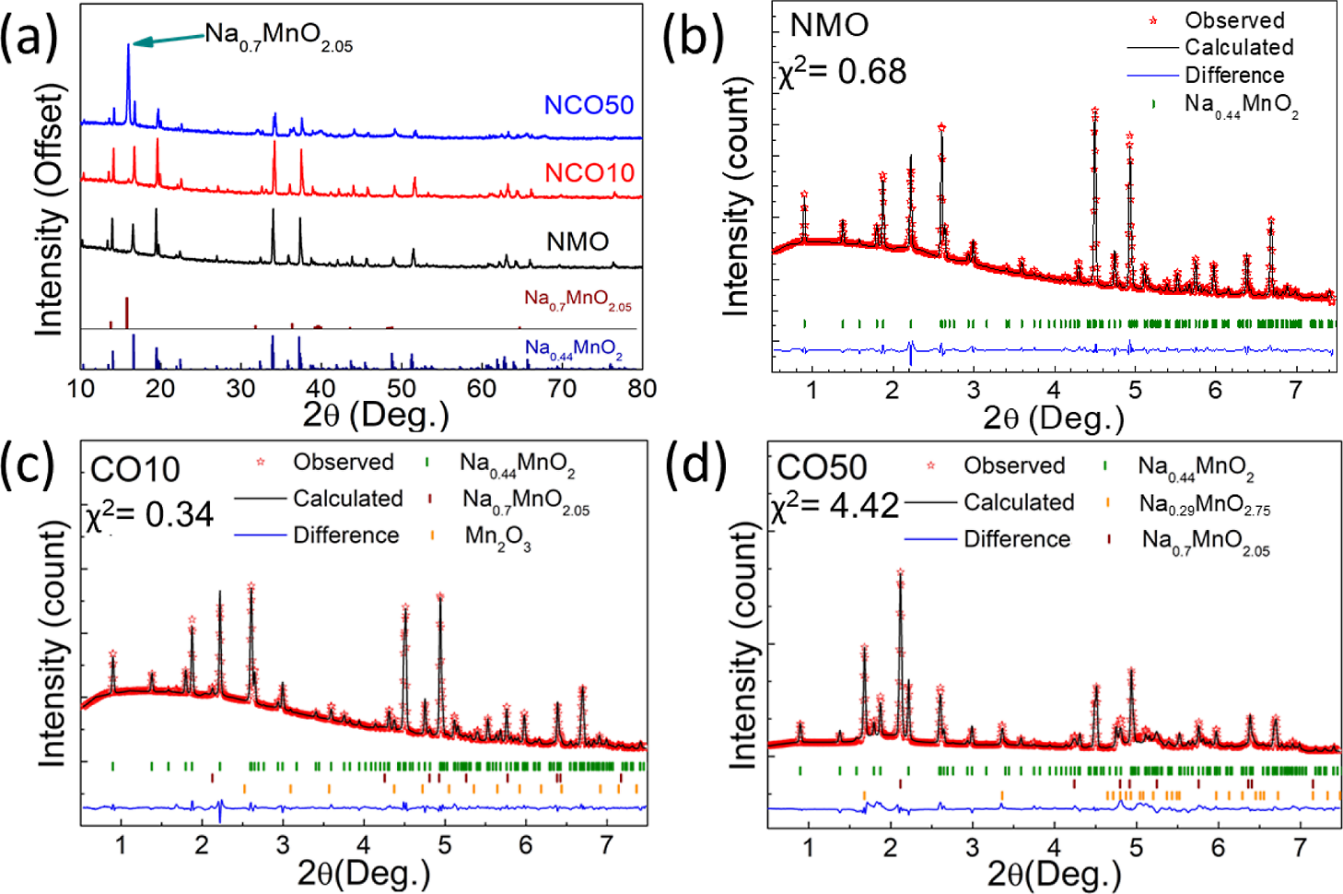
(a) Comparison of the offset XRD results of samples. Rietveld analysis of (b) NMO, (c) NCO10, and (d) NCO50 samples using synchrotron X-ray powder diffraction data.

The powder XRD patterns of the samples obtained using a Cu Kα source between 10 and 80° 2θ angles are shown in Figure 1a. While no impurity phase is observed in the NMO sample, the Na_0.7_MnO_2.05_ phase is present in CO10 and CO50 samples, which is also observed in Na > 0.6 structures in previous studies.^36,37^ For detailed crystal structural analysis, synchrotron powder XRD experiments were performed, and the refined results of all samples are shown in Figure 1b–d. The NMO crystal structure belongs to the *Pbam* space group^38^ (JCPDS no. 27-0750) and does not contain any impurity phase (Figure 1b). The results show that increasing Co substitution causes the formation of the P2 phase in the CO10 sample (Figure 1c). The P2 phase is in the form of Na_0.7_Mn_0_O_2.05_ with the *P*6_3_/*mmc* space group (JCPDS no. 27-0751).^39^ In this sample, a small amount (8%) of Mn_2_O_3_ impurity is detected. Further increase in the Co substitution leads to the formation of a second layered structure Na_0.29_MnO_2.75_, with the space group *C*12/*m*1(JCPDS no. 43-1456) in the CO50 sample. The refined lattice parameters of the phases in NMO, CO10, and CO50 are listed in Table S1. In addition, inductively coupled plasma–mass spectrometry (ICP–MS) elemental analysis results can be seen in Table S2.

**Figure 2:**
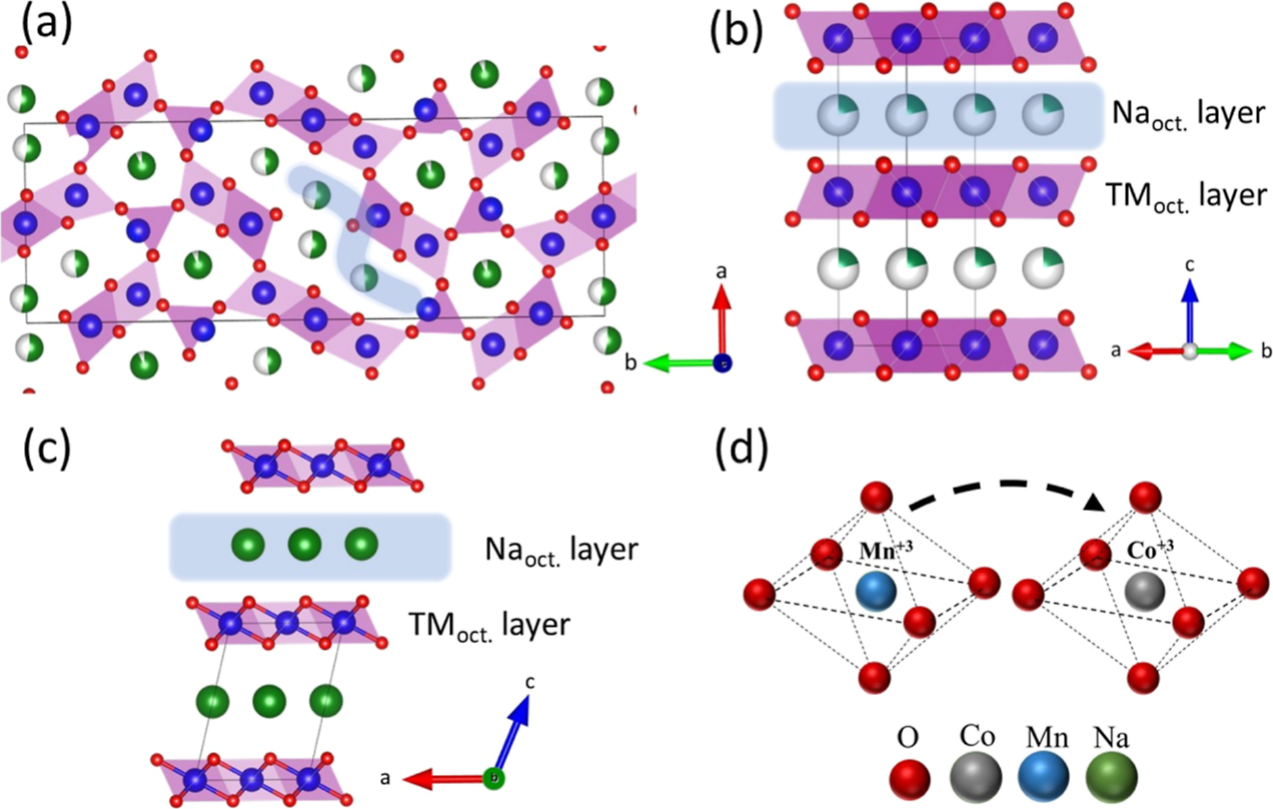
Crystal structure of (a) tunnel-type Na_0.44_MnO_2_ and layered (b) *P*6_3_/*mmc* Na_0.7_MnO_2.05_ and (c) *C*12/*m*1 Na_0.29_MnO_2.75_. (d) Illustration of substitution of Co^3+^ for Mn^3+^ in the octahedral structure.

The crystal structure of Na_0.44_MnO_2_ consists of MnO_5_ square pyramids and MnO_6_ octahedra that are arranged to create two distinct tunnel structures, one of which is larger with an S-shape, while the other is smaller (Figure 2a).^40^ The Na ions in the S-shaped tunnels participate in redox reactions, while those in the small tunnels are not mobile. In addition, the Mn^4+^ ions are located in the MnO_6_ octahedral sites; while half of the Mn^3+^ ions are located in the MnO_5_ square pyramids, the other half are in the MnO_6_ octahedra.^41^ Na_0.7_MnO_2.05_ (Figure 2b) and Na_0.29_MnO_2.75_ (Figure 2c) consist of the distribution of the [Mn^3+^O_6_]^3–^ and [Mn^4+^O_6_]^2–^ structures into transition-metal-oxide layers.^42,43^ The Na_0.7_MnO_2.05_ structure consists of MnO_2_ layers and the Na^+^ ions between them. Under normal conditions, a fully stoichiometric NaMnO_2_ structure is expected, however, the Na vacancies cause the formation of the Na-deficient Na_0.7_MnO_2.05_ structure with a vacancy in every six MnO_6_ octahedra, as reported by Parant et al.^44^ In addition, the Mn ions are distributed in the crystal structure with a ratio of 60% Mn^3+^ and 40% Mn^4+^.^45^ Similarly, the Na_0.29_MnO_2.75_ phase with the layered structure has Na vacancies between the layers and is possible to show high battery performance as other layered structures. The CO10 and CO50 samples have Mn^4+^ and Mn^3+^ sites where substitution of Co ions is possible. However, the ionic radii of Mn^3+^ and Mn^4+^ are 0.58 and 0.53 Å, respectively, while the ionic radius of Co^3+^ is 0.54 Å.^46^ Therefore, Co ions are more likely to substitute for Mn^3+^ sites (Figure 2d). The atomic positions and occupations of all samples obtained as a result of structural refinement can be seen in Tables S3, S4, and S5.

The Mn^3+^ ions in the structure of NMO and the change in lattice parameters indicate that a similar substitution may be possible for this sample. Table S1 shows the decrease in *a* and *c* parameters with increasing Co substitution. Notably, the reduction in the *c* parameter can be elucidated by the smaller atomic radius of the Co^3+^ ion.^32^ The lattice parameter *a* of the P2 Na_0.7_MnO_2.05_ phase exhibited a decrease as the Co substitution increased, whereas the *c* parameter showed an increase. Moreover, a reduction in the unit cell volume was observed. This can be attributed to the smaller size of the Co^3+^ ion compared to the Mn^3+^ ion as mentioned above. Furthermore, the shorter Co–O bond in comparison to the Mn–O bond^47^ likely led to the shrinking of the (Mn/Co)O_6_ octahedra, thereby resulting in an expansion of the *d*-space along the *c*-axis in the P2 structure. The distortion index values of the Na_0.7_MnO_2.05_ c/a ratio, as presented in Table S1, further demonstrate that lattice distortion (*c*/*a*) increases with increasing Co substitution. A comparative analysis of lattice distortion ratios with Co substitution reveals that the *c*/*a* ratio of the tunnel Na_0.44_MnO_2_ sample increased from 0.3103 in the NMO sample to 0.3110 in the CO50 sample. Similarly, the *c*/*a* ratio of the Na_0.7_MnO_2.05_ sample increased from 3.8805 in the CO10 sample to 3.9189 in the CO50 sample. Based on these findings, the observed increase in lattice distortion amounts to 2.2% in the tunnel Na_0.44_MnO_2_ sample, while it reaches 9.8% in the P2 Na_0.7_MnO_2.05_ sample. The higher lattice distortion rates in the P2 structure indicate that Co^3+^ ions tend to substitute Mn^3+^ sites in the P2 structure instead of in the tunnel structure.

**Figure 3:**
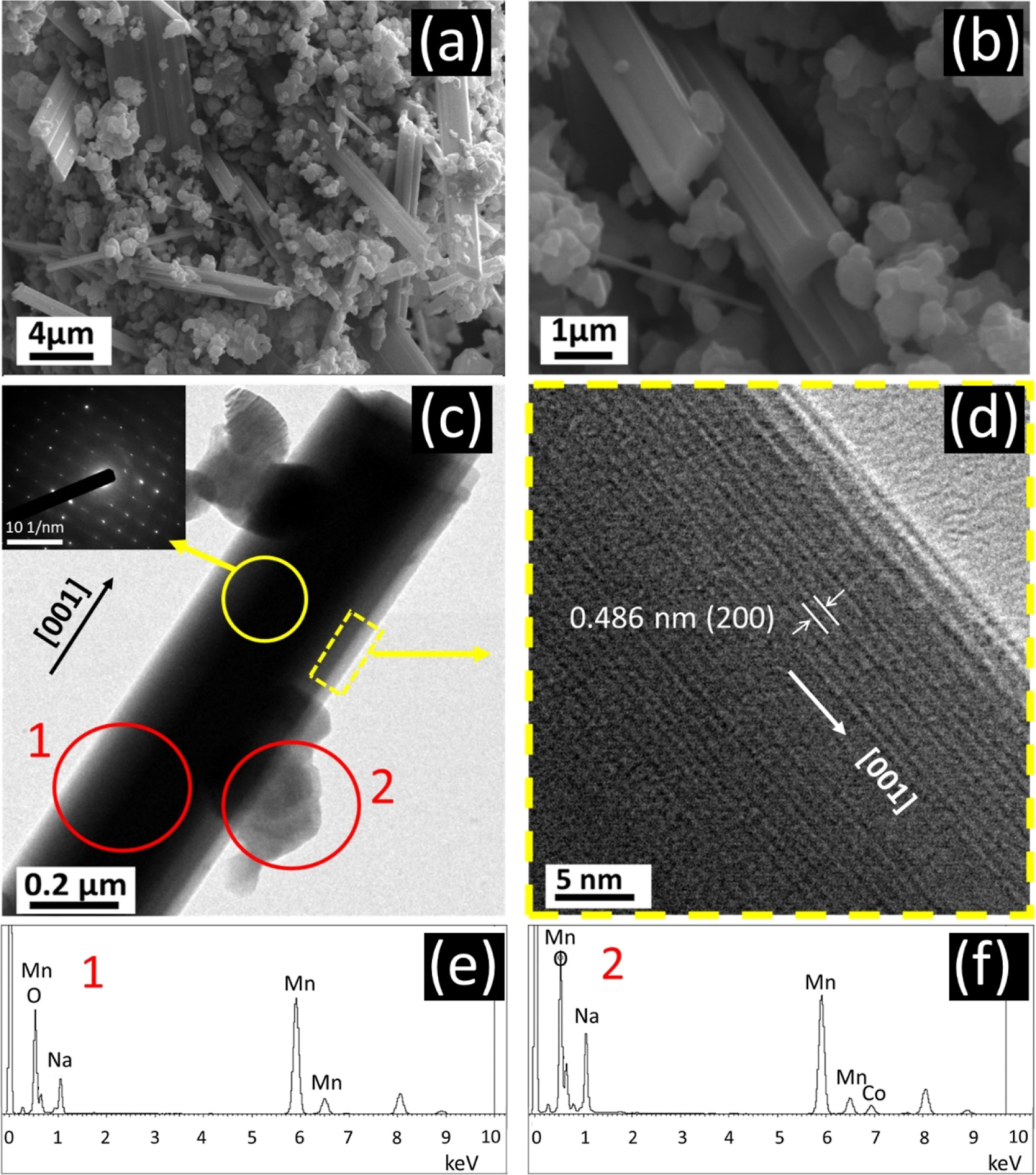
(a,b) SEM image of CO50 under different magnifications. (c) TEM image and SAED pattern (inset) and (d) HRTEM image of the CO50 sample. EDS spectrum of red-circled regions in the TEM image of the (e) tunnel structure and (f) P2 structure.

Figure 3a,b shows the SEM images of the CO50 sample. The rod-like and granular structures are homogeneously distributed in the NMO sample, and no distinct features are observed. The thickness of rod-like structures is ∼250 nm. The P2 phase has a uniform particle distribution, but locally agglomerated structures are also observed. The average size of the particles in the granular structure is ∼250 μm. The nanorod-like crystal structure dominates the particle distribution in the NMO and CO10 samples (Figure S1).

TEM measurements were performed for detailed crystal structure analysis (Figure 3). Both rod and P2 structures that determine the morphology of CO50 can be seen in Figure 3c. The rod structure has a diameter of ∼0.5 μm, while the granular P2 structure has a thickness of ∼0.2 μm. The SAED image given as the inset in Figure 3c indicates the single crystal structure of the rod structure. The HRTEM image of the rod particle shows a uniform lattice fringe with a *d*-spacing of 0.486 nm (Figure 3b), corresponding to the interplanar spacing distance of the (200) plane of the tunnel structure.^48^ TEM, HRTEM, and SAED images of NMO and CO10 are presented in Figure S2. The EDS results of the compositions of the rod (1) and P2 (2) structures reveal the tendency of Co to substitute Mn sites in the P2 structure instead of the rod structure.

**Figure 4:**
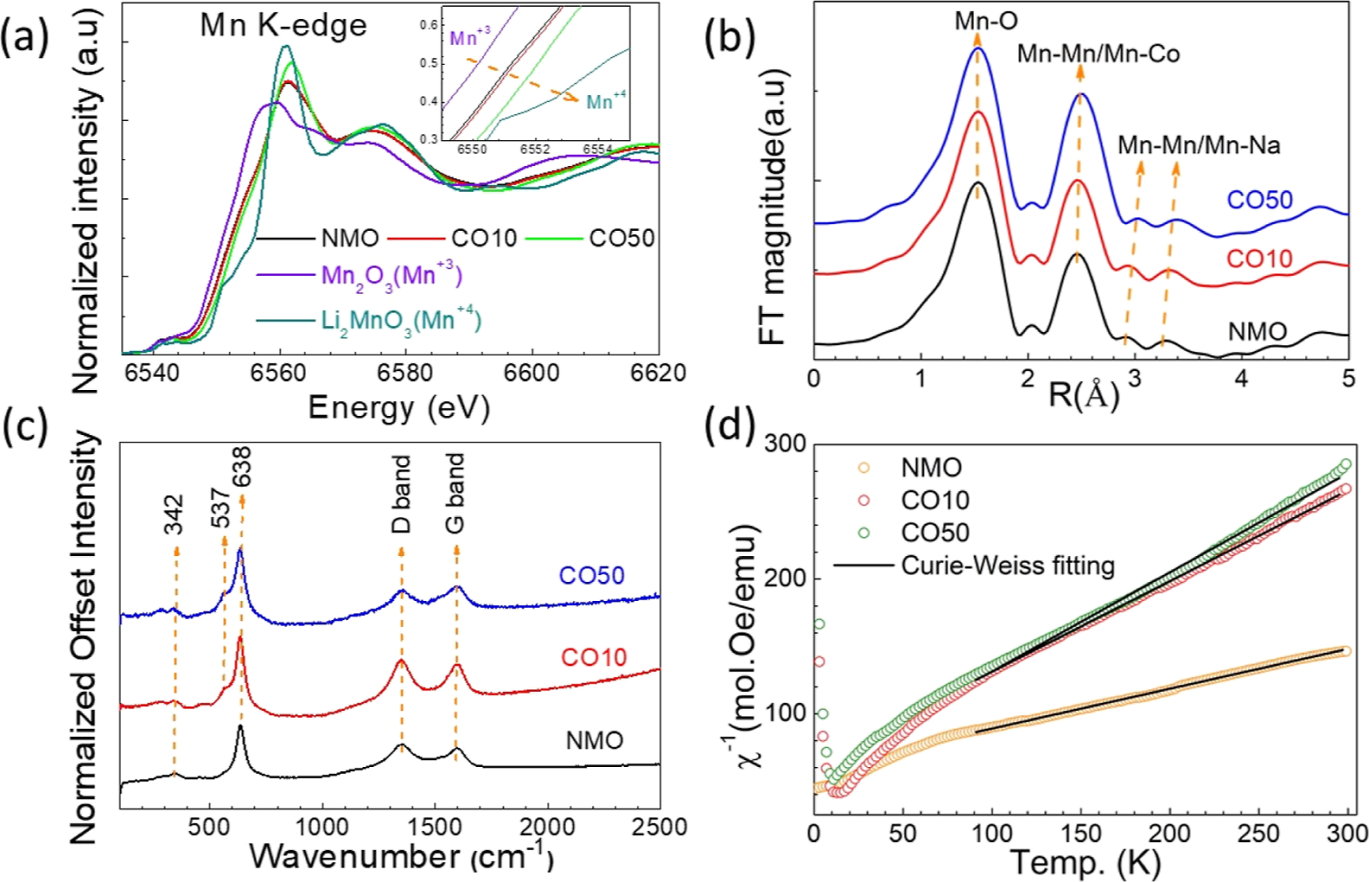
(a) Mn-K edge spectra of the samples and of Mn_2_O_3_ and Li_2_MnO_3_ with Mn^3+^ and Mn^4+^ references and energy shift of the samples from Mn^3+^ to Mn^4+^ (inset). (b) Fourier transformed Mn EXAFS spectra (k-weight = 3) in R space. The peaks correspond to scattering from the nearest neighbor’s atoms. (c) Average Raman spectra of samples. D band and G band shifts come from the carbon used in electrodes. (d) Fitted inverse magnetic susceptibility of samples by the Curie–Weiss law between 100 and 300 K as a function of temperature.

EXAFS measurements were performed to investigate the changes in the local structures and the valence state of Mn upon Co substitution. Normalized XANES curves are shown in Figure 4a. MnO_2_ and Li_2_MnO_3_ are used as Mn^3+^ and Mn^4+^ standards, respectively. It is evident that all the samples contain both Mn^3+^ and Mn^4+^ since their XANES curves comprise a combination of the two. The shift of the curves to higher energies with increasing Co substitution reveals an increase in the Mn^4+^/Mn^3+^ ratio (Figure 1a inset). For further analysis, the changes in the nearest neighborhoods of Mn can be examined by applying the Fourier transform (FT) to the Mn K-edge EXAFS data. The peaks in Figure 4b represent the radial distances to the nearest neighbors of Mn where the Mn–O distance decreases with Co substitution. There are two possible explanations for this phenomenon. The first is related to the distortion in the MnO_6_ octahedral structure. The second reason is that the Mn^4+^–O bond length is shorter than that of Mn^3+^–O^49^ as a result of the increased Mn^4+^/Mn^3+^ ratio upon Co substitution.

The Raman spectra of samples in the form of electrodes are shown in Figure 4c. In all samples, the defective/disordered carbon D-band and G-band at 1352 and 1593 cm^–1^, respectively, are observed due to the carbon used to prepare the electrodes. Apart from these two carbon-induced bands, the strongest band peak is seen at 638 cm^–1^ and is attributed to the Mn–O band, which is caused by the symmetric stretching of the MnO_6_ octahedral structure.^39^ The vibration band at 342 cm^–1^ is attributed to M–O (M = Mn and Na) bending, and the peak has both Na–O and O–Mn–O bending bands.^39^ The shoulder at 527 cm^–1^ starts with the Co substitution and represents the Co–O band.^50^

Magnetization measurements have the potential for revealing significant information regarding the substitution of Co for Mn sites. We know that Co tends to incorporate into the P2 structure instead of the rod structure from morphology analysis. Mn atoms are in the MnO_6_ octahedral environment in the P2 structure. In this system, Mn atoms can have Mn^3+^ low-spin (LS) or high-spin (HS) and Mn^4+^ spin configurations. Mn^3+^ has theoretical effective magnetic moments of 2.83 and 4.90 μ_B_ in LS and HS configurations, respectively, while Mn^4+^ has 3.97 μ_B_. On the other hand, Co has the Co^3+^ spin configuration in the octahedral system and has effective magnetic moments of 0 and 4.90 μ_B_ in the LS and HS spin states, respectively.^51^ The variation of the inverse magnetic susceptibility of the samples as a function of temperature is shown in Figure 4d. By fitting the magnetization curves according to the Curie–Weiss law, the effective magnetic moments obtained for NMO, CO10, and CO50 are 3.76, 3.44, and 3.26 μ_B_, respectively. The reduced effective magnetic moment upon Co substitution indicates the only possibility that the Co^3+^ ions are in LS configuration.

**Figure 5:**
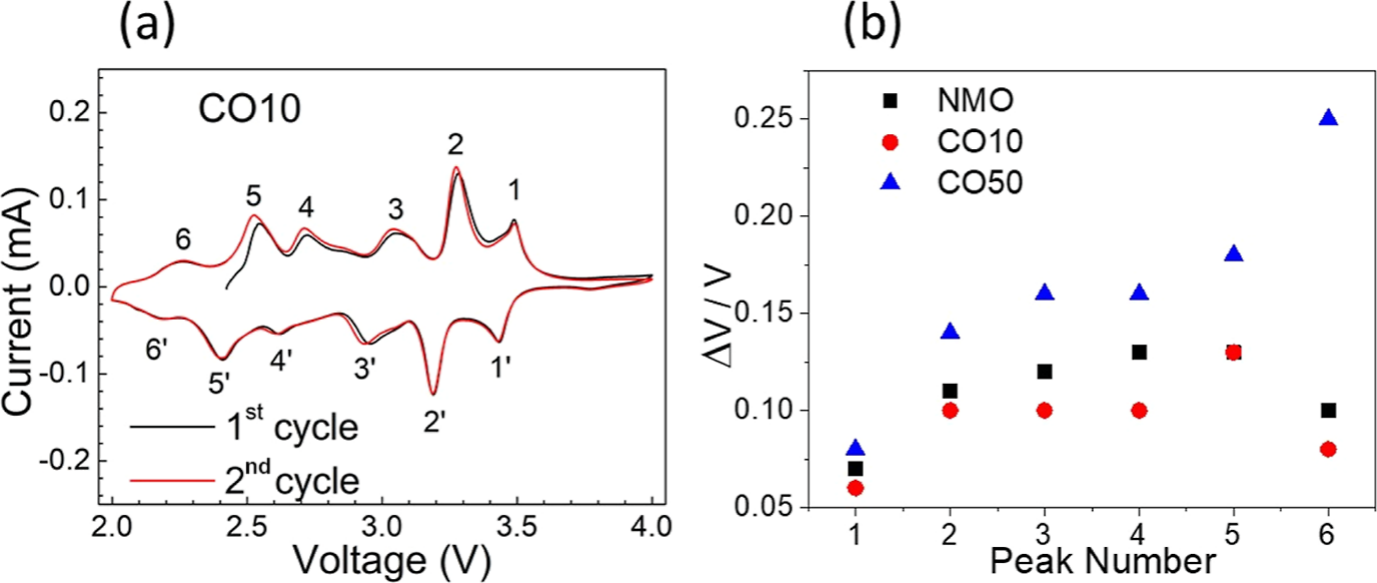
(a) CV measurements of the CO10 sample. The numbers with an apostrophe (‘)label the cathodic reaction peaks. (b) Potential difference between the anodic and cathodic peaks for NMO, CO10, and CO50.

Figure 5a shows first two CV curves of CO10 and the voltage polarization of all samples. The six peaks in the CV curves visible in the anodic and cathodic scans indicate a complex multiphase transition mechanism during Na-ion insertion/extraction processes, consistent with the six biphasic transitions reported previously.^52^ In their detailed study, Sauvage et al. stated that these biphasic transformations do not indicate the formation of a new structure but a transition between very similar structures. Although these biphasic transitions cannot be precisely identified, it is possible that they are caused by the interaction of Na^+^ with Mn^4+^ and Mn^3+^ at different sites during intercalation/deintercalation. In addition, biphasic transitions at a low potential are attributed to the redox process of Na ions in large S structures, whereas transitions at a high potential are attributed to Na redox processes in small tunnel structures.^53^ The appearance of the same redox peaks in the second voltammetric cycle indicates that the reactions are reversible. CV curves of NMO and CO50 samples can be seen in Figure S3.

The difference between cathodic and anodic peaks is an indication of voltage polarization (Δ*V*).^54^ The results obtained from the CV data show that the highest Δ*V* is at CO50 and that CO10 and NMO have similar polarizations (Table S6) (Figure 5b). The increase in polarization can be delivered as one factor that negatively affects the discharge capacity. As seen below, the capacity performance test results correlate with this assumption.

**Figure 6:**
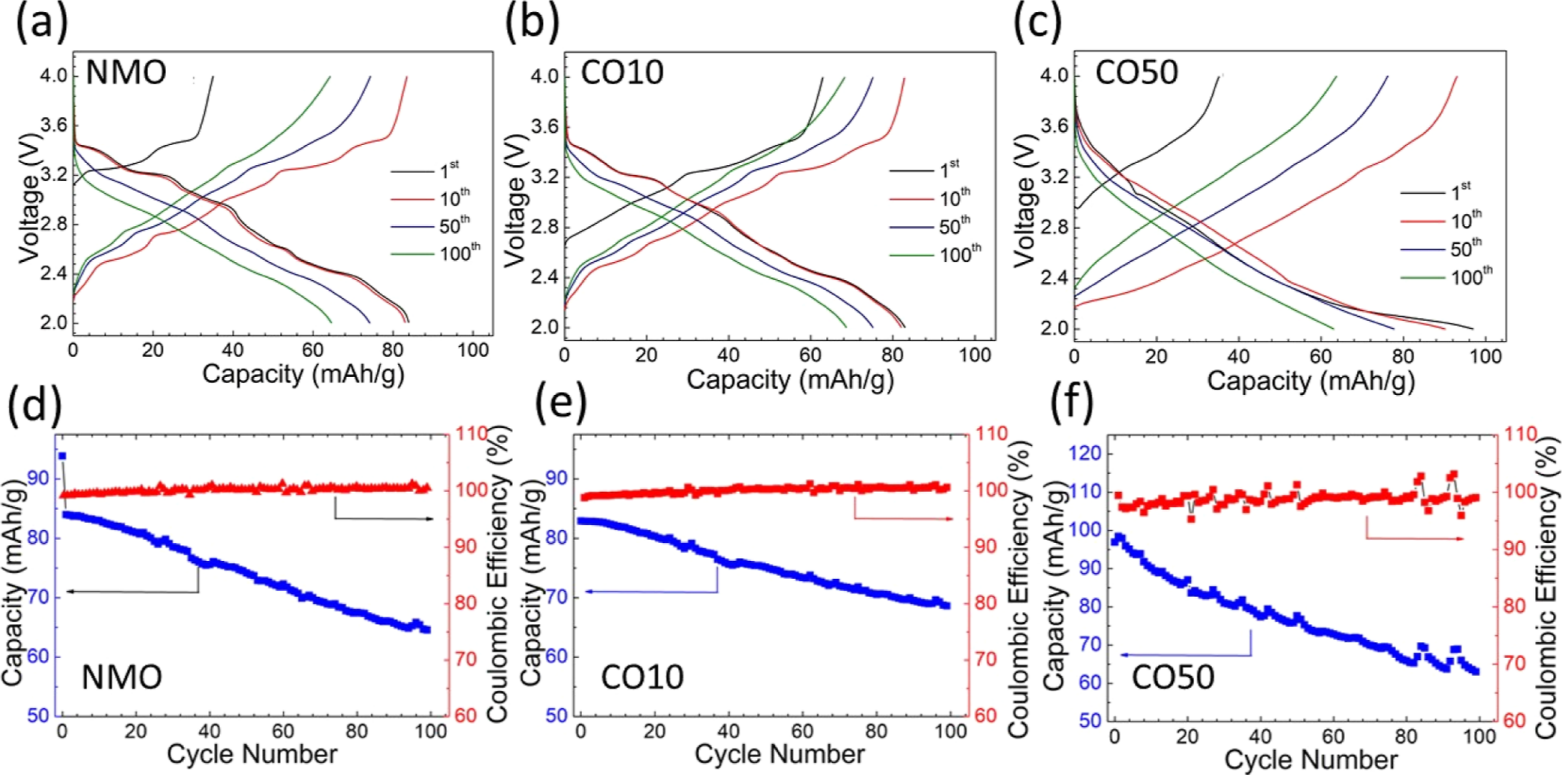
Constant current charge/discharge profile (a–c) and cyclic performance and Coulombic efficiency (d–f) in the 2.0–4.0 V voltage window at 0.3 C current rate for the NMO, NCO10, and NCO50 samples.

Figure 6 shows the samples’ voltage–capacity curves, cyclic performances, and Coulombic efficiencies. Series phase transitions explain the multiple plateaus seen in the NMO sample during Na insertion/extraction reactions (Figure 6a). Similar phase transition plateaus are also observed in the CO10 sample (Figure 6b), which agrees with the CV results. On the other hand, CO50 charge/discharge curves are smoother (Figure 6c), indicating that phase transitions are suppressed. The dominant P2–Na_0.66_MnO_2_ phase in the CO50 sample is responsible for the suppression of the phase transformation. However, the effect of the Mn^4+^/Mn^3+^ redox peaks of the P2-type layered Na_0.7_MnO_2.05_ phase can be seen as broad and intense peaks in the 2.0–2.4 V range.^55^

The cyclic performance tests and Coulombic efficiency results of the samples in the 2.0–4.0 V range at 0.3 C are shown in Figure 6d–f. The initial discharge capacities of NMO, CO10, and CO50 are 83.86, 82.91, and 98.92 mA h/g, respectively. These results are quite remarkable compared to the initial capacity results of previous studies synthesized by the solid-state reaction method.^41^ Furthermore, the capacity retentions are 77, 84, and 63% for NMA, CO10, and CO50, respectively, after 100 cycles at 0.3 C current rate, which are significantly higher than the reported counterparts. Sauvage et al. reported the initial discharge capacity of NMO synthesized by the solid-state reaction method as 80 mA h/g with a 50% capacity loss after 50 cycles at 0.1 C rate.^56^ Furthermore, Wang et al. stated an initial capacity of 164 mA h/g at a current density of 0.1 A/g for Na_0·7_MnO_2.05_, but the capacity decreases to 48 mA h/g (30% capacity retention) after 200 cycles.^57^ In our samples, the Coulombic efficiencies remain stable for 100 cycles.

**Figure 7:**
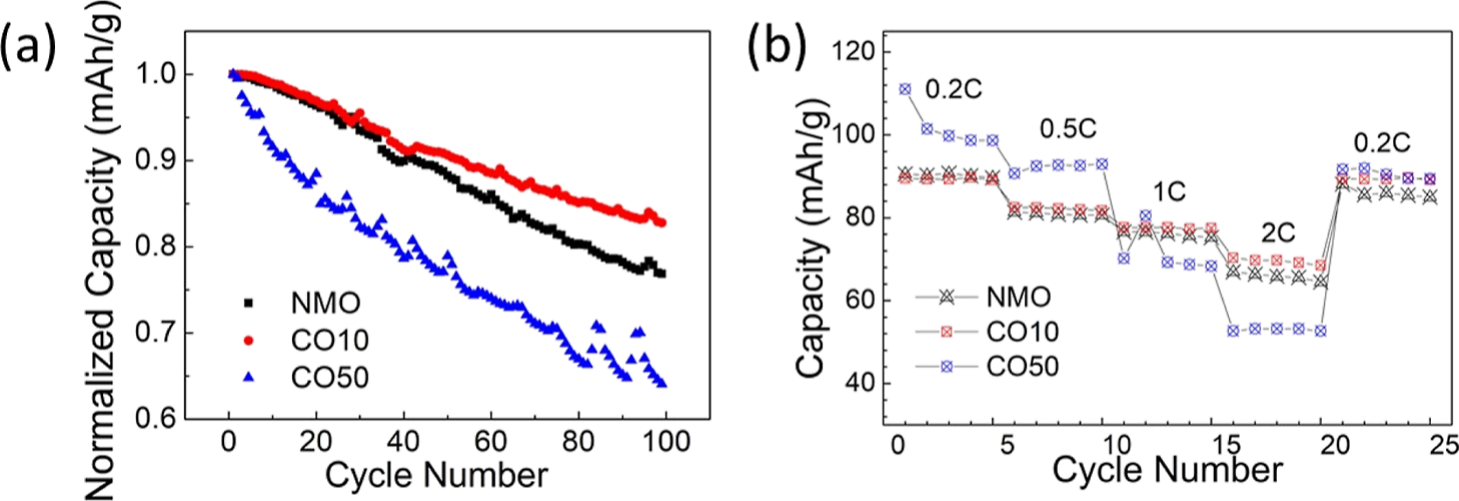
Normalized capacity as a function of cycle number (a) and rate capability of the samples (b).

To compare the capacity retention of the samples, their normalized specific capacities were evaluated (Figure 7a). The fact that CO10 has the lowest polarization may explain its high-capacity retention. Figure 7b shows the rate capability of the samples tested at different current densities. Although the discharge capacities of CO10 and NMO samples at 0.2 C, 0.5 C, and 1 C current densities are similar, the discharge capacity of the CO10 sample is slightly higher at 2 C current rate. However, while the discharge capacity of C50 is higher at 0.2 C and 0.5 C current densities, it has an inferior discharge capacity at 2 C than the other samples. The current rate was reduced from 2 C to 0.2 C to observe if the samples can recover their initial capacities at 0.2 C. While the CO50 sample shows a 10% capacity loss, NMO and CO10 reach their initial capacity at 0.2 C rate, indicating structural stability in these two samples.

## 4 Conclusions

In this study, composite cathode materials with tunnel-P2 structures were obtained by substituting Co for Mn sites to prevent the rapid structural degradation of the tunnel-type NMO material. The structures of NMO and Co-substituted CO10 and CO50 samples were identified by synchrotron powder XRD. The results show that Co substitution causes the formation of the P2 phase in the CO10 sample and P2 phases in the CO50 sample alongside the tunnel structure of NMO. XANES measurements show that the Mn^4+^/Mn^3+^ ratio increases with increasing Co substitution, reducing the Jahn–Teller effect and suppressing the structural degradation. Morphological characterizations show that the substituted Co ions are located at the Mn sites in the P2/P3 structure rather than the tunnel structure. The electrochemical tests show the CO10 composite material has 84% capacity retention after 100 cycles at 0.3 C rate, indicating its potential for Na-ion battery applications due to its high cycling performance.

## Supporting Information Available

Refined lattice parameters of all phases, SEM images of NMO and CO50 samples, TEM images of NMO and CO10 samples, and first two CV cycles of NMO and CO50 samples (PDF)
