# Supplementary material for: Tunnel/Layer Composite Na0.44MnO2 Cathode Material with Enhanced Structural Stability via Cobalt Doping for Sodium-Ion Batteries
Source: ACS Omega. 2023 Jul 22;8(30):27170–8. doi: 10.1021/acsomega.3c02315 (PMC10399157; doi:10.1021/acsomega.3c02315)
Supplement: Supplementary file 1 — ao3c02315_si_001.pdf [file ao3c02315_si_001.pdf]

Supporting Information

Tunnel/Layer Composite $Na_{0.44}MnO_2$ Cathode Material with Enhanced Structural Stability via Cobalt Doping for Sodium-Ion Batteries

Erdinc Oz[*†‡], *Serdar Altin*[§], *Sevda Avci*[‖]

[†] Physics Department, Ataturk University, Erzurum, 25400, Turkey

[‡] Nanoscience and Nanoengineering Department, Ataturk University, Erzurum, 25400, Turkey

[§] Physics Department, Inonu University, Malatya, 44210, Turkey

[‖] Department of Engineering Physics, Istanbul Medeniyet University, Istanbul, 34700, Turkey

* Corresponding author: erdinc.oz@atauni.edu.tr

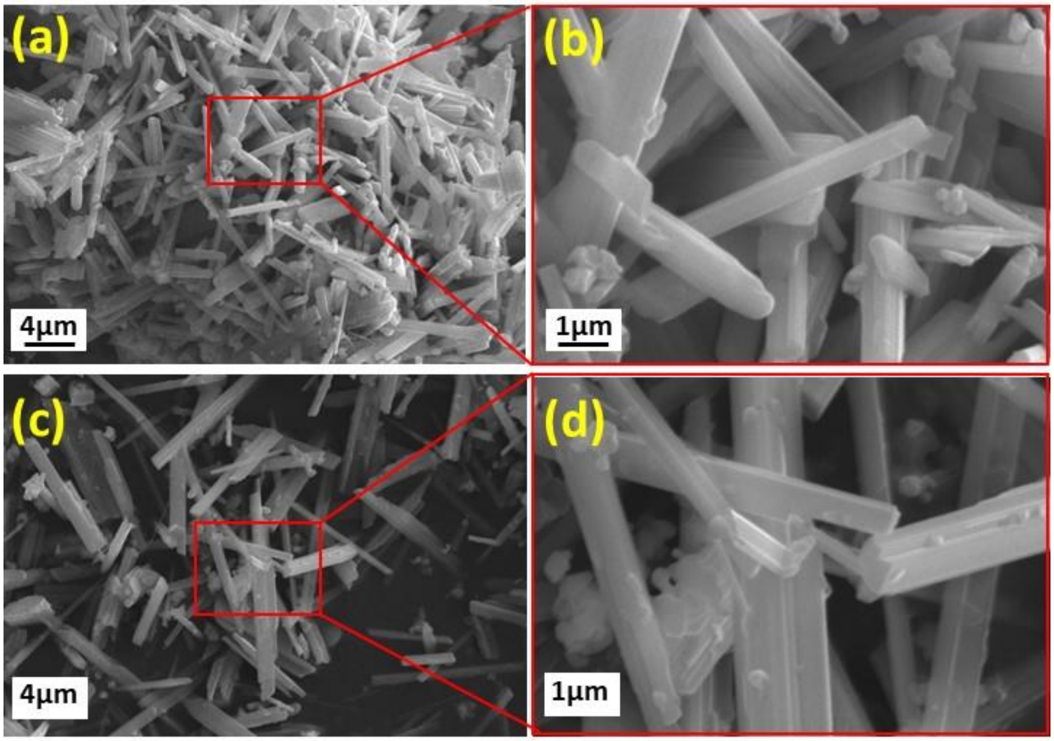

Figure S1 SEM image in different magnetizations of (a)-(b) NMO and (c)-(d) CO10 samples.

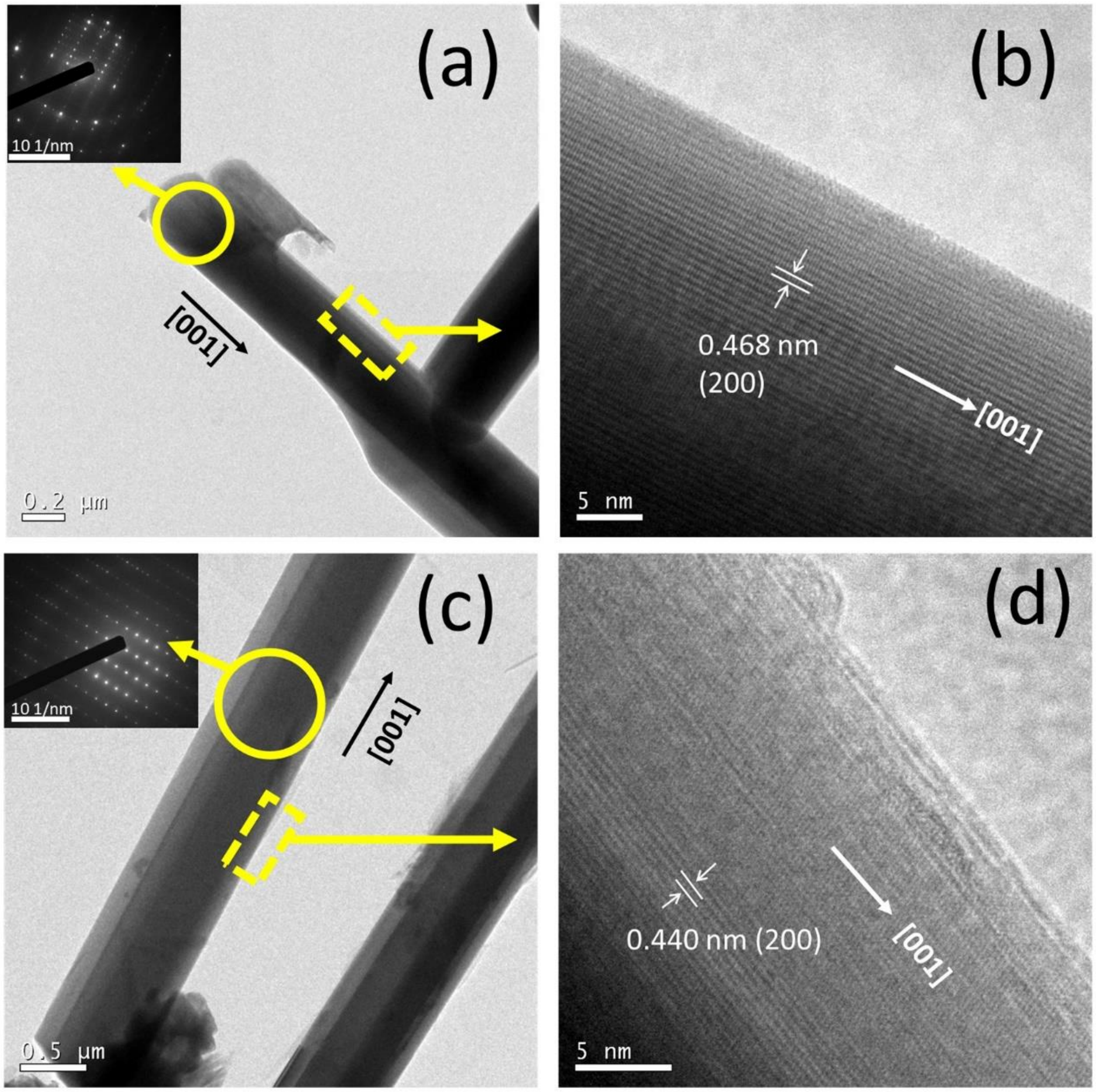

Figure S2 TEM and HRTEM image of rod-like structures in (a)-(b) NMO, (c)-(d) CO10 samples.

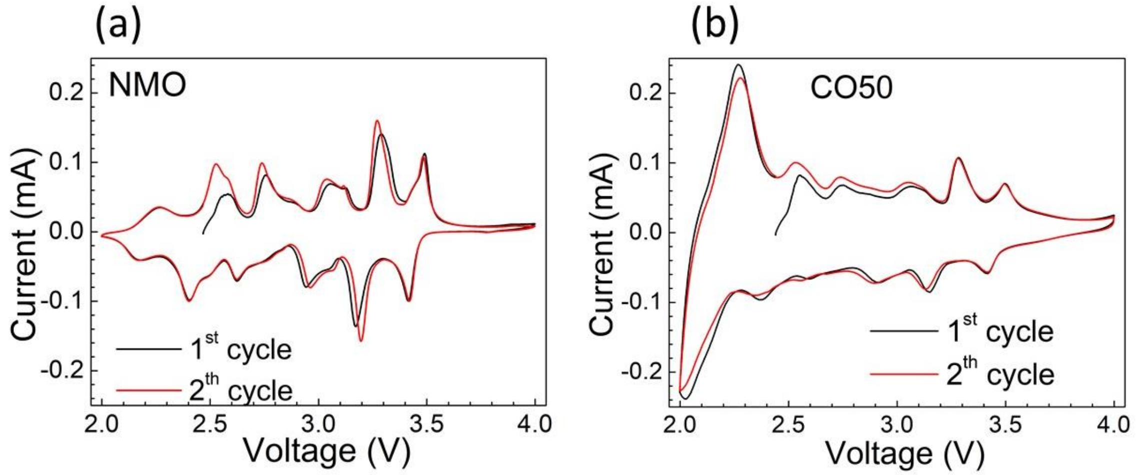

Figure S3 Cyclic voltammetry curves of (a) NMO and (b) CO50 samples.

Table S1 Phase fractions, lattice parameters, and refinement constant of samples.

Sample	Phase Fractions (%)	Lattice Parameters (Å)	R_{wp}	Volume (Å^3)	*c*/*a*
**NMO**	$Na_{0.44}MnO_2$ (%100)	*a*=9.1321, *b*=26.4474, *c*=2.8339	7.90	684.474	0.3103
**CO10**	$Na_{0.44}MnO_2$ (%72.25)	*a*=9.0995, *b*=26.4320, *c*=2.8287	6.79	680.359	0.3109
	$Na_{0.7}MnO_{2.05}$ (%25.84)	*a*=*b*=2.8700, *c*=11.1373	6.86	79.447	3.8805
	Mn_2O_3 (%1.90)	*a*=*b*=*c*= 9.4112	9.11	833.550	1
**CO50**	$Na_{0.44}MnO_2$ (%49.70)	*a*=9.0954, *b*=26.45012, *c*=2.8284	7.12	680.277	0.3110
	$Na_{0.7}MnO_{2.05}$ (%36.45)	*a*=*b*=2.8564, c=11.1941	7.92	79.100	3.9189
	$Na_{0.29}MnO_{2.75}$ (%13.84)	*a*=5.4367, *b*=3.0270, *c*=7.2639	5.19	116.096	1.3361

Table S2 Comparison of theoretical and ICP-MS weight percentage results of the samples.

Sample	Elements	Theoretical (%)	Elements	Experimental (%)
**NMO**	Na	10.42	Na	9.12
	Mn	56.61	Mn	57.04
	Co	-	Co	-
**CO10**	Na	10.42	Na	9.21
	Mn	56.01	Mn	65.51
	Co	0.61	Co	0.76
**CO50**	Na	10.40	Na	8.98
	Mn	53.67	Mn	56.70
	Co	3.03	Co	3.01

Table S3 Atomic positions and occupations of the NMO sample calculated by refinement analysis.

$Na_{0.44}MnO_2$, Space group: *Pbam*,				
**Atom**	**x**	**y**	**z**	**occupation**
Mn1	0.86798	0.19339	0.50000	0.50000
Na2	0.67588	0.08677	0.50000	0.31249
Na3	0.14205	0.00274	0.00000	0.27950
Na1	0.21686	0.20042	0.00000	0.45566
Mn2	0.50000	0.00000	0.00000	0.25000
Mn3	0.53344	0.19334	0.00000	0.50000
Mn4	0.36290	0.08886	0.50000	0.50000
Mn5	0.01508	0.10992	0.00000	0.50000
O1	0.95799	0.06615	0.50000	0.50665
O2	0.93956	0.23381	0.00000	0.49486
O3	0.04403	0.16198	0.50000	0.51818
O4	0.51169	0.07200	0.00000	0.49299
O5	0.22557	0.09872	0.00000	0.46233
O6	0.37061	0.00498	0.50000	0.48215
O7	0.41842	0.16847	0.50000	0.47688
O8	0.66553	0.21681	0.50000	0.47765
O9	0.83120	0.14379	0.00000	0.47556

Table S4 Atomic positions and occupations of the phases in CO10 sample were calculated by refinement analysis.

$Na_{0.44}MnO_2$, Space group: *Pbam*,				
**Atom**	**x**	**y**	**z**	**occupation**
Mn1	0.86641	0.19294	0.50000	0.49029
Na2	0.68617	0.08781	0.50000	0.33440
Na3	0.12442	0.00269	0.00000	0.22866
Na1	0.22063	0.20662	0.00000	0.40017
Mn2	0.50000	0.00000	0.00000	0.25465
Mn3	0.54351	0.19435	0.00000	0.49243
Mn4	0.36271	0.08947	0.50000	0.47940
Mn5	0.01709	0.11034	0.00000	0.50082
O1	0.95859	0.06838	0.50000	0.54339
O2	0.92798	0.23437	0.00000	0.52435
O3	0.04662	0.15916	0.50000	0.48514
O4	0.49913	0.06500	0.00000	0.48996
O5	0.22619	0.09911	0.00000	0.47214
O6	0.37455	0.00236	0.50000	0.48071
O7	0.40917	0.17021	0.50000	0.49737
O8	0.66694	0.20665	0.50000	0.47178

O9	0.82925	0.13942	0.00000	0.51528

$Na_{0.7}MnO_{2.05}$, Space group: *P 63/m m c*,

**Atom**	**x**	**y**	**z**	**occupation**
Mn1	0.0000	0.00000	0.50000	0.06068
Na2	0.66667	0.33333	0.25000	0.10021
Na1	0.00000	0.00000	0.25000	0.06321
O1	0.33333	0.66667	0.06457	0.19280

Mn_2O_3, Space group: *I a -3*,

**Atom**	**x**	**y**	**z**	**occupation**
Mn1	0.00000	0.00000	0.00000	0.06068
Mn2	0.28000	0.00000	0.25000	0.50000
O1	0.09425	0.33387	0.14819	1.84521

Table S5 Atomic positions and occupations of the phases in CO50 sample calculated by refinement analysis.

$Na_{0.44}MnO_2$, Space group: *Pbam*,				
**Atom**	**x**	**y**	**z**	**occupation**
Mn1	0.86798	0.00000	0.50000	0.500
Na2	0.67588	0.19334	0.50000	0.312
Na3	0.14205	0.08886	0.00000	0.280
Na1	0.21686	0.10992	0.00000	0.456
Mn2	0.50000	0.06722	0.00000	0.250
Mn3	0.53344	0.23538	0.00000	0.500
Mn4	0.36290	0.15931	0.50000	0.500
Mn5	0.01508	0.07187	0.00000	0.500
O1	0.96882	0.09870	0.50000	0.670
O2	0.92123	-0.00142	0.00000	0.601
O3	0.04388	0.16446	0.50000	0.606
O4	0.50573	0.21342	0.00000	0.528
O5	0.21940	0.14826	0.00000	0.639
O6	0.37520	0.00000	0.50000	0.613
O7	0.43744	0.19334	0.50000	0.532
O8	0.68116	0.08886	0.50000	0.549
O9	0.83822	0.10992	0.00000	0.645

$Na_{0.7}MnO_{2.05}$, Space group: *P 63/m m c*,				
**Atom**	**x**	**y**	**z**	**occupation**
Mn1	0.0000	0.00000	0.50000	0.08300
Na2	0.66667	0.33333	0.25000	0.02700
Na1	0.00000	0.00000	0.25000	0.01800
O1	0.33333	0.66667	0.08800	0.16700

$Na_{0.29}MnO_{2.75}$, Space group: *C12/m1*,				
**Atom**	**x**	**y**	**z**	**occupation**
Mn1	0.00000	0.00000	0.00000	0.25000
Na1	0.59500	0.00000	0.50000	0.06900
O1	0.38966	0.00000	0.11657	0.50000
O2	0.59500	0.00000	0.50000	0.15000
O3	0.00000	0.00000	0.50000	0.05757

Table S6 Cathodic and anodic peak positions of samples.

Peak Number	Anodic Peak Positions (V)			Cathodic Peak Positions (V)			ΔV ($V_{Anodic–Cathodic}$)		
	NMO	CO10	CO50	NMO	CO10	CO50	ΔV_{NMO}	ΔVC_{O10}	ΔV_{CO50}
1	3.49	3.49	3.50	3.42	3.43	3.42	0.07	0.06	0.08
2	3.28	3.28	3.28	3.17	3.18	3.14	0.11	0.10	0.14
3	3.05	3.04	3.06	2.93	2.94	2.90	0.12	0.10	0.16
4	2.75	2.71	2.74	2.62	2.61	2.58	0.13	0.10	0.16
5	2.53	2.53	2.54	2.40	2.40	2.36	0.13	0.13	0.18
6	2.26	2.26	2.27	2.16	2.18	2.02	0.1	0.08	0.25
